# Amniotic Membrane Extract Protects Islets From Serum-Deprivation Induced Impairments and Improves Islet Transplantation Outcome

**DOI:** 10.3389/fendo.2020.587450

**Published:** 2020-12-08

**Authors:** Zhaoming Yang, Xiaohang Li, Chengshuo Zhang, Ning Sun, Tingwei Guo, Jianzhen Lin, Feng Li, Jialin Zhang

**Affiliations:** Department of Hepatobiliary Surgery, The First Hospital of China Medical University, Shenyang, China

**Keywords:** amniotic membrane extract, serum-deprivation, apoptosis, islet transplantation, type 1 diabetes

## Abstract

Islet culture prior to transplantation is a standard practice in many transplantation centers. Nevertheless, the abundant islet mass loss and function impairment during this serum-deprivation culture period restrain the success of islet transplantation. In the present study, we used a natural biomaterial derived product, amniotic membrane extract (AME), as medium supplementation of islet pretransplant cultivation to investigate its protective effect on islet survival and function and its underlying mechanisms, as well as the engraftment outcome of islets following AME treatment. Results showed that AME supplementation improved islet viability and function, and decreased islet apoptosis and islet loss during serum-deprived culture. This was associated with the increased phosphorylation of PI3K/Akt and MAPK/ERK signaling pathway. Moreover, transplantation of serum-deprivation stressed islets that were pre-treated with AME into diabetic mice revealed better blood glucose control and improved islet graft survival. In conclusion, AME could improve islet survival and function *in vivo* and *in vitro*, and was at least partially through increasing phosphorylation of PI3K/Akt and MAPK/ERK signaling pathway.

## Introduction

Islet transplantation is an effective β-cell replacement therapy that could help type 1 diabetes mellitus (T1DM) patients to achieve euglycemia (1). However, shortage of pancreas donors, the side effect of immunosuppression, and poor graft survival are some of the limitations related to this therapy (2, 3). Besides, early graft loss and the instant blood mediated inflammatory reaction (IBMIR) restrain the success of islet transplantation (4, 5). It is known that the achievement of insulin independence and the long-term outcome of islet transplantation are also determined by the quality and quantity of the initially transplanted islets (6).

Originally, islet culture is not included in the Edmonton Protocol, which required isolated islets to be infused within 4 h (2, 7). This causes some difficulties in recipient preparation and islet quality control, and increases the risk of operation. Therefore in clinical islet transplantation, after isolation and purification, islets are cultured *in vitro* for 24–72 h (8–10) before transplantation to enable the quality control of the isolated islets, the pretreatment of recipients, and the transportation of islets to remote transplant centers (11, 12), and a 48-h cultivation tend to be the standard practice accepted by most islet transplantation centers (13, 14). Nevertheless, islet viability, β-cell function were impaired, and approximately 10–20% of islet mass was lost during this period, which exacerbates the islet shortage and restrains the success of islet transplantation (15). Fetal bovine serum (FBS) is ideal for culturing islets, because it contains abundant growth factors and extracellular matrix components (16). However in clinical islet transplantation, human serum albumin (HSA) is used as a supplementation of the culture media (17) instead of FBS to avoid animal-derived factors, which causes a serum-deprived condition that may impair islet viability and function (18, 19). Therefore, it’s urgent to explore strategies to prevent islet apoptosis during serum-deprived cultivation *in vitro*.

Human amniotic membrane (hAM), a commonly discarded tissue after Caesarean Section, has been utilized as a clinical therapeutic biomaterial for more than a century. Over recent years, several studies have investigated homogenates or extracts of amniotic membrane (AME) reporting them to contain abundant biomolecules that promote wound healing and tissue regeneration (20, 21), reduce inflammation and prevent oxidative stress-induced injury (22, 23), and promote cell proliferation, including epidermal growth factor (EGF), hepatocyte growth factor (HGF), heavy chain hyaluronic acid in complex with pentraxin 3 (HC-HA/PTX3), tissue inhibitor of metalloproteinases (TIMPs), IL-1 receptor antagonist (IL1RA), trombospondin-1 (TSP-1), Fas ligand (FasL), fibronectin, and collagen types I, III, IV, and V (24). Due to these properties and its low immunogenicity, AME has been used clinically in the treatment of acute or chronic chemical burns (25). Based on these findings, we hypothesize that amniotic membrane extract, containing abundance of growth factors and extracellular matrix components, can protect islets against serum deprivation induced impairments.

In this study, amniotic membrane extract was prepared and characterized, and its potential to protect islets during *in vitro* culture was examined. Specifically, amniotic membrane extract was added to the islet culture medium, and the viability, apoptosis, percent loss, and glucose-stimulated insulin secretion (GSIS) function of serum-deprived islets were evaluated. Cultured islets were further transplanted in the renal subcapsular space of syngenic diabetic recipients to assess the potential of AME treated islets in the regulation of blood glucose.

## Methods and Materials

### Animals

Male C57BL/6 mice were obtained from Liaoning Changsheng biotechnology co., Ltd (Benxi, China). Six-to-eight-week old C57BL/6 mice (weighing 20–22 g), housed under Specific pathogen-free (SPF) conditions and fed *ad libitum* with food and water, were used as donor mice and diabetic recipients. All the surgeries were performed under isoflurane (RWD, Shenzhen, China) inhalation anesthesia. This study was conducted in accordance with the National Institute of Health Guide and Use of Laboratory Animals and was approved by the Animal Care and Use Committee of China Medical University.

### Preparation and Characterization of Amniotic Membrane Extract

All amniotic membranes were collected from the Department of Obstetrics of the First Affiliated Hospital of China Medical University. The study was approved by the Ethical Committee of the First Affiliated Hospital of China Medical University ([2019]2019-219-3), and informed consent was signed by all the participants choosing the elective caesarean section. Each prospective donor was screened for infectious diseases, and those who tested negative for HIV, hepatitis B, hepatitis C, cytomegalovirus, and syphilis were included in this study.

Amniotic membrane extract was prepared according to the methods described by Ebrahimi et al. (20). with some modifications. Briefly, hAM was washed with PBS (SH30256.01, Hyclone, USA) that contained 1,000 U/ml penicillin and 0.1 mg/ml streptomycin (pen/strep, P1400, Solarbio) three times to remove blood clots and was then cut into small pieces. Next, hAM was submerged in liquid nitrogen and manually ground into a fine powder. After weighing, they were mixed with PBS at a ratio of 1:1 (w/v), after which the mixture was homogenized by a sonicator (F6/10, Jingxin, Shanghai, China) at 10,000 rpm on ice for 1 h. The homogenate was centrifuged at 4,000 g at 4°C for 10 min, and the supernatant was collected and re-centrifuged at 15,000 g at 4°C for 5 min. The final supernatant was collected and filtered through a 0.22-μm filter.

Amniotic membrane was collected from 12 healthy donors and divided into four batches for the preparation of AME. The total protein in each batch of AME was assessed using a standard Bradford protein assay (P0006, Beyotime) according to the manufacturer’s instructions. The concentrations of TIMP-1, EGF, HGF, and IL-1RA, as important amniotic membrane proteins for maintaining islet vitality (26–33), were assessed using commercially available ELISA kits (DEG00, DHG00, DTM100, and DRA00B, R&D System Inc., Minneapolis, USA). The stability of the growth factors were assayed in freshly extracted AME and after 7 days to 1 month of storage at −80°C. The concentration of growth factors was also compared between cryopreserved AME (prepared 3 and 6 months ago respectively) and freshly prepared AME.

### Isolation and Culture of Islets

Isolation of mouse islets was previously described (34). Briefly, the common bile duct was cannulated with a 31 G steel needle, and the pancreas was inflated with 2–3 ml collagenase V solution (1 mg/ml) (C9263, Sigma-Aldrich). Then, the perfused pancreas was acquired and digested in a 37°C thermostatic water bath for 20 min. Subsequently, the digestion was terminated with precooled Hank’s balanced salt solution (HBSS) (14025134, Gibco, USA) containing 10% fetal bovine serum (FBS) (10099141, Gibco, Australia) with vigorous agitation, and islets were purified on Ficoll gradients (density 1.108, 1.096, 1.069, and 1.037 g/ml) (F2637, Sigma-Aldrich).

Purified islets were collected and cultured in RPMI 1640 (Gibco, CA, USA) containing 100 U/ml penicillin and 0.01 mg/ml streptomycin (P1400, Solarbio) at 37°C in a humidified atmosphere consisting of 95% air and 5% CO_2_. For the serum-deprivation condition, islets were cultured in medium supplemented with 0.625% bovine serum albumin (BSA, A1933, Sigma-Aldrich) as previously described (33). To test the effects of AME on islet during cultivation, medium containing 0.625% BSA plus 0.1, 0.5, 1.0, 1.5 mg/ml AME was used to islet culture. Islets cultivated in medium containing 10% FBS (10099141, Gibco, Australia) were used as the positive control group.

### Assessment of Islet Viability

After 48 h of cultivation, islets were assessed for viability using acridine orange (AO, Sigma, USA) and ethidium bromide (EB, Sigma, USA) as previously described (35). Briefly, 100 mg/ml acridine orange and 100 mg/ml ethidium bromide in PBS were mixed at a ratio of 1:1 to generate the working solution. The working solution was added to the islets containing medium (20 μl working solution per ml medium), and after 2–5 min incubation at room temperature, islet viability was examined by an inverted fluorescent microscope (Nikon Corporation). Viable cells were stained green, and viability was calculated as the percentage of the viable cells to total cells.

### Percent Islet Recovery

To determine islet yield, islets prior to and post 48-h culture were harvested and counted as previously described (36). Briefly, aliquots from both experimental and control groups were stained with dithizone (Sigma Aldrich, USA) and counted. The ratio of total islets harvested 48 h post-culture relative to the number of islets harvested prior to culture was defined as the percentage of islet recovery.

### Assessment of Islet Apoptosis

Apoptosis of the cultured islets was assessed by terminal deoxynucleotidyl transferase-mediated dUTP nick end labeling (TUNEL) assay (TUNEL)staining and flow cytometry as previously described (36–38). For TUNEL staining analysis, briefly, islets were collected and fixed in 4% paraformaldehyde, embedded in 3% agar (A8190, Solarbio) to create a solid cellular-matrix interaction, then processed and embedded in paraffin. After deparaffinization and antigen heat retrieval, islet sections were washed with phosphate-buffered saline (PBS) supplemented with 5% bovine serum albumin (BSA, Solarbio), followed by incubation with guinea pig anti-insulin (ab7842, Abcam, UK) primary antibodies at 1:100 overnight at 4°C. Then, samples were rinsed in PBS and incubated with goat anti-guinea pig (Alexa 488, Beyotime, Shanghai, China) secondary antibodies at 1:500 for 60 min at room temperature. Next, fluorescein isothiocyanate-dUTP with TdT enzyme (G3250, Promega, USA) was added and counterstained with the DAPI (Solarbio) in antifade mounting medium (Solarbio). Apoptosis was calculated as the percentage of the TUNEL-stained cells to the both insulin and nuclei positive cells using ImageJ software (downloaded from the NIH website [https://imagej.nih.gov/ij/]).

In addition, islet apoptosis was evaluated by flow cytometry using TUNEL Assay Kit - BrdU-Red (ab66110, Abcam) according to the manufacturer’s instructions. After 48-h cultivation, 75–100 islets from respective samples were hand-picked, washed with PBS twice, and dispersed into single cells by 0.1 mg/ml bovine trypsin (Sigma-Aldrich, USA) and 2 mmol/l EDTA in PBS for 5 min at 37°C. Cells were then fixed with 4% formaldehyde and incubated for 30 min at room temperature. After being washed with PBS, cells were incubated in 70% ethanol for 30 min at 4°C. Subsequently, cells were washed and incubated in DNA Labeling Solution for 60 min at 37°C. After incubated in antibody solution for 30 min at room temperature in dark, 7-AAD/RNase A solution was added and cells were incubated for 30 min at room temperature in dark. Finally, cells were analyzed by flow cytometry (FACSCalibur; Becton Dickinson).

### Glucose Stimulated Insulin Secretion Assay

After 48 h of cultivation, islets were collected and assessed for secretion function. Ten islets were preincubated in Krebs-Ringer-bicarbonate buffer (KRBB) containing 2.8 mM glucose for 30 min. After preincubation, islets were incubated in KRBB containing 2.8 mM glucose (basal insulin secretion) for 1 h and in KRBB containing 16.8 mM glucose for 1 h (GSIS) to collect the supernatants. Secreted insulin was determined using the Ultrasensitive Mouse Insulin Elisa kit (10-1249-01, Mercodia, Sweden). The stimulation index was calculated as the ratio of GSIS to basal insulin secretion (39).

### Western Blot

Total cell extracts were analyzed by Western blot as previously described (40). Briefly, the total protein from treated cells (200 islets) was extracted using RIPA lysis buffer (P0013B, Beyotime, Shanghai, China) supplemented with protease inhibitors (P1006, Beyotime) and phosphatase inhibitors (P1046, Beyotime). Then, the protein concentration was determined using the BCA protein assay kit (P0010S, Beyotime). Twenty micrograms of total protein extracts were resolved by 10 or 12% SDS-PAGE (KGP113K, KeyGen Biotech. Co. Ltd., Nanjing, China) and transferred to PVDF membranes (Millipore, Temecula, CA, USA). Subsequently, membranes were blocked with 5% non-fat dried milk solubilized in TBST for 2 h and probed with primary antibodies against p-AKT (Ser473) (4060, Cell Signaling Technology), AKT (9272, Cell Signaling Technology), p-ERK1/2 (9101, Cell Signaling Technology), ERK1/2 (9102, Cell Signaling Technology), Bcl2 (26593-1-AP, Proteintech), BAX (50599-2-Ig, Proteintech), Cleaved Caspase-3 (9664, Cell Signaling Technology), and β-actin (AF0003, Beyotime) at 4°C overnight. Finally, proteins were visualized using the ECL Western blot protocol (P0018, Beyotime) after incubation with the secondary antibodies for 2 h at room temperature. The intensity of bands was measured using the Image Lab 5.0 software.

### *In Vivo* Islet Transplantation Underneath the Kidney Capsule

Diabetic recipients were induced by intraperitoneal injection of streptozotocin (S0130, Sigma-Aldrich) at 180 mg/kg in sodium citrate buffer (0.1 mol/L, pH 4.5) (Solarbio, Beijing, China). Recipients with non-fasting blood glucose exceeding 19.4 mmol/L for 2 consecutive days were considered diabetic. After 48-h cultivation under different conditions (0.625% BSA as BSA only group; 0.625% BSA + 0.5 mg/ml AME as BSA + AME group; 10% FBS as FBS group), a marginal mass of islets (200 islets) was handpicked and transplanted underneath the kidney capsule of recipient mice under the anaesthetized condition. After islet transplantation, recipient mice were measured for non-fasting blood glucose using ACCU-CHEK^®^ Performa glucometer (Roche, USA) every 3 days until the end of the study. Reversal of diabetes was defined as two consecutive readings of <11.1 mmol/L. After the 6-week observation period, islet grafts were totally removed after reversal of diabetes by resection of the left kidney to confirm the graft dependent euglycemia. On the contrary, loss of graft function was defined as the non-fasting blood glucose exceeding 19.4 mmol/L for two consecutive readings. To confirm the graft-dependent euglycemia, nephrectomies were performed at the end of the study and non-fasting blood glucose was measured for 7 days to verify the recurrence of hyperglycemia.

### Intraperitoneal Glucose Tolerance Test

To evaluate the metabolic function of the cultured islets *in vivo*, glucose tolerance tests were performed at 30 days post-transplantation. Following fasting overnight, glucose bolus (2 g/kg) was intraperitoneally injected to recipient mice. Blood glucose was measured at 0, 15, 30, 60, 90, 120 min after injection. Overt diabetic mice were excluded in this experiment.

### Histological Analysis

Graft was retrieved at 42 days after transplantation (41). The samples were fixed, processed, embedded, sliced, and subjected to HE, immunohistochemistry, and immunofluorescence staining as previously described (42). Sequentially sliced sections were either stained with hematoxylin and eosin (HE) (Servicebio) or treated with immunohistochemistry or immunofluorescence staining. For immunohistochemistry staining, paraffin-embedded tissue sections were deparaffinized and hydrated using xylene and gradient of alcohol to water. Next, samples were blocked and incubated with insulin (ab7842, Abcam) primary antibody following antigen retrieval and endogenous peroxidase activity quenching. Finally, samples were incubated with the secondary antibody (Proteintech) and detected with the DAB Horseradish Peroxidase Color Development Kit (Beyotime) and counterstained with hematoxylin (MX Biotechnologies). Images were captured by light microscopy (Nikon, Japan). For immunofluorescence staining, samples were deparaffinized and after antigen retrieval and blocking by 5% bovine serum albumin (BSA, w/v; Solarbio), samples were incubated with insulin (ab7842, Abcam, UK) and glucagon (2760, Cell Signaling Technology, USA) primary antibodies at 1:100 at 4°C overnight. Then samples were washed with PBS and incubated with the secondary antibodies consisting of goat anti-guinea pig (Alexa 488, Beyotime, Shanghai, China) at 1:500 and goat antirabbit (Alexa 555, Beyotime) at 1:500. Finally, samples were counterstained with DAPI (Beyotime) in antifade mounting medium (Solarbio) and the images were acquired using a fluorescence microscope (Nikon, Japan).

### Graft Insulin Content

After retrieval, islet bearing kidneys were stored at −80°C until insulin analysis. Graft insulin content was measured, as previously described (43). Briefly, islet grafts were homogenized and sonicated at 4°C in 10 ml of 2 mmol/L acetic acid containing 0.25% BSA for 2 h. Next, homogenates were sonicated again and centrifuged at 10,000 g for 25min at 4°C, and the supernatant was collected. A total of 5 ml acetic acid was added to the remaining pellets and extracted again by sonication and centrifugation, and the second supernatant was collected. Total volume was measured by a combination of the two supernatants, and the samples were assayed for insulin content using the Ultrasensitive Mouse Insulin Elisa kit (10-1249-01, Mercodia, Sweden).

### Statistical Analysis

Statistical analysis was performed using Graph-Pad Prism 6 software (GraphPad Software, Inc., USA). Data are expressed as the mean ± standard deviations. The differences between groups were analyzed by Student’s unpaired t-test with two-tailed *P*-values and one-way ANOVA, followed by Tukey’s multiple comparisons test. A *P*-value of <0.05 was considered a statistically significant difference. All experiments were performed at least three times.

## Results

### Effects of AME on Serum-Deprived Islet Viability and Recovery *In Vitro*

**Figure 1:**
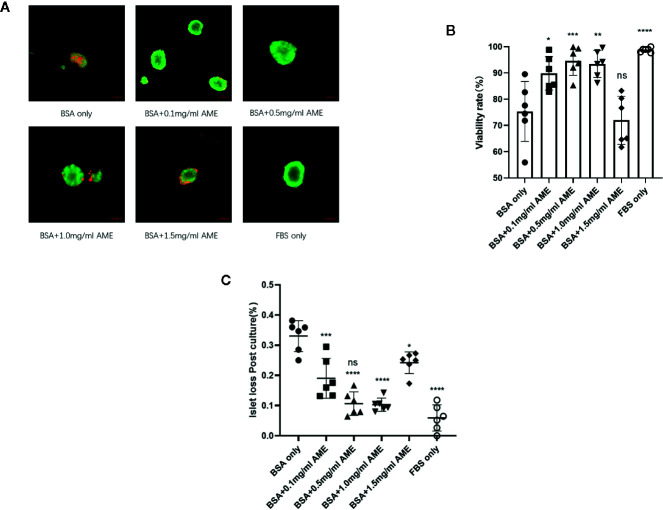
AME significantly increased the viability and recovery of serum-deprived islets **(A)**. Representative images are showing AO/EB staining of isolated islets after 48 h cultivation. Viable cells are stained as green **(B)**. The percentage of viable cells was significantly increased with AME treatment (*p < 0.05, **p < 0.01, ***p < 0.001, ****p < 0.0001 and ns *vs.* BSA only group, One-way ANOVA). Results are shown as means ± SD of six independent experiments, with 75–100 islets/condition for each independent experiment. Scale bar: 100 μm **(C)**. The percentage of islet loss post 48-h cultivation *in vitro* (***p < 0.001, ****p < 0.0001, ****p < 0.0001, *p < 0.05, and ****p < 0.0001 *vs.* BSA only group, and ns *vs.* FBS only group, One-way ANOVA). Results are shown as means ± SD of six independent experiments, with 75–100 islets/condition for each independent experiment.

In order to investigate whether AME affects the viability of serum-deprived islets, 0.1–1.5 mg/ml AME were applied to islet culture media. After 48 h of cultivation, serum deprivation induced a significant decrease in cell viability compared to the whole serum group. Different concentrations (0.1–1.0 mg/ml) of AME protected islets against serum deprivation-induced impairment and maintained cell viability after a 48-h *in vitro* culture, while 1.5 mg/ml AME showed no protective effects (**Figures 1A, B**). Additionally, islets were quantified to determine islet yield after 48 h cultivation. Results showed that serum-deprivation induced a significant islet loss (33.05 ± 5.11%, BSA only group), while medium supplementation of AME ameliorated serum-deprivation induced islet loss (10.30 ± 2.19 *vs*. 5.91 ± 4.32%, BSA + 0.5 mg/ml AME *vs.* FBS only, *p* > 0.05) (**Figure 1C**).

### Apoptosis Rate Evaluated by TUNEL Staining and Flow Cytometry

**Figure 2:**
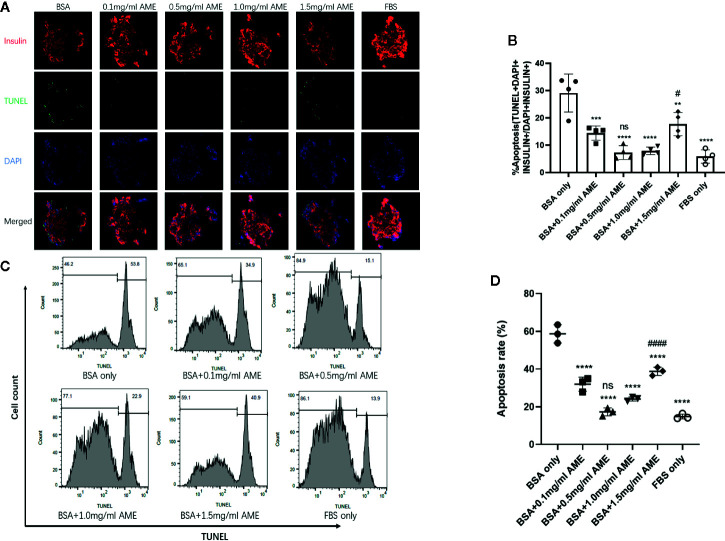
AME significantly decreased the apoptosis rate of serum-deprived islets **(A)**. Representative images are showing TUNEL staining of isolated islets after 48 h cultivation. Insulin (red), apoptosis (green), and nucleus/DAPI (blue) were stained for the analysis **(B)**. The percentage of apoptosis was calculated as TUNEL+DAPI+Insulin+/DAPI+Insulin+. (**p < 0.01, ***p < 0.001, and ****p < 0.0001 *vs.* BSA only group. ^#^p < 0.05 vs. BSA + 0.5 mg/ml AME, and ns *vs.* FBS only group, One-way ANOVA). Results are shown as means ± SD of four independent experiments, with 15–20 islets/condition for each independent experiment **(C)**. Detection of DNA fragmentation (TUNEL staining) by flow cytometry **(D)**. The percentage of apoptotic cells determined by flow cytometry following trypsin digestion. (****p < 0.0001 *vs.* BSA only group. ^####^p < 0.0001 *vs.* BSA + 0.5 mg/ml AME group, and ns *vs.* FBS only group, One-way ANOVA). Results are shown as means ± SD of three independent experiments.

TUNEL staining was performed to evaluate the apoptosis rate of cultured islets in different groups. Results showed that AME protected islets from serum deprivation-induced impairment and significantly decreased apoptosis rate. We found that supplementation of AME at the concentration of 0.5 mg/ml mostly benefited the cultured islets, and there was no significant difference between groups of 0.5 mg/ml AME and FBS only. Though 1.5 mg/ml AME obviously decreased cellular apoptosis rate, its apoptosis rate was significantly higher than that in the 0.5 mg/ml group (**Figures 2A, B**). In addition, a flow cytometry analysis was conducted to further verify islet cell apoptosis. There was a high level of DNA fragmentation (TUNEL positive staining) primarily due to the process of trypsin digestion. Results showed that a significant increase in DNA fragmentation was observed in serum-deprivation group compared to FBS only group (p < 0.001), while cell death was not significantly increased in islets cultured in medium supplemented with 0.5 mg/ml AME group. Islets cultured in medium supplemented with 0.1, 1.0, and 1.5 mg/ml AME also showed significant decreases in apoptosis (p < 0.001) compared to serum-deprivation group (**Figures 2C, D**).

### Effects of AME on GSIS Function of Serum-Deprived Islet *In Vitro*

**Figure 3:**
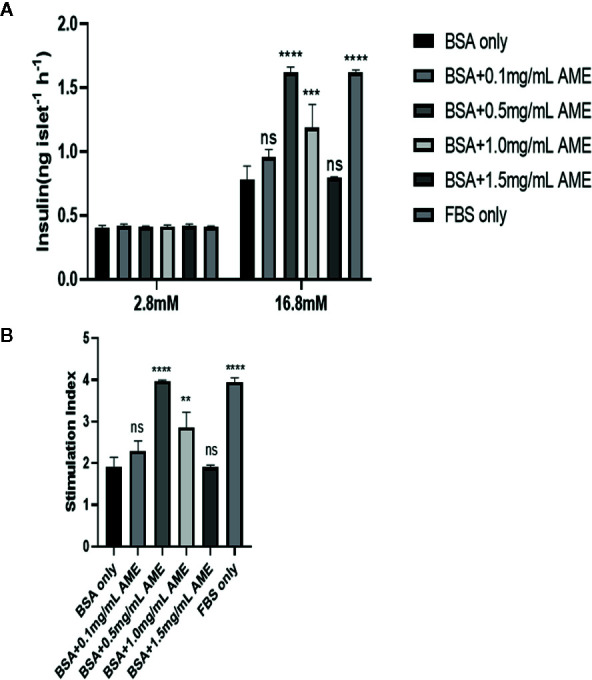
Glucose-stimulated insulin release assay and stimulation index analysis **(A)**. After 48 h cultivation, 10 islets were picked from each group and assayed for GSIS. The improvement of insulin secretory function by AME peaked at the concentration of 0.5 mg/ml (***p < 0.001, ****p < 0.0001, and ns *vs.* BSA only group, Two-way ANOVA) **(B)**. The stimulation index was calculated (**p < 0.01, ****p < 0.0001, and ns *vs.* BSA only group, One-way ANOVA). Results are shown as means ± SD of three independent experiments.

To assess whether AME had a further protective effect on islet insulin secretory function *in vitro*, glucose-stimulated insulin secretion assay was performed after 48 h cultivation. Results showed that AME improved serum-deprived islet insulin secretory function at concentrations of 0.5 mg/ml (p < 0.0001) and 1.0 mg/ml (p = 0.0002) under high glucose (16.8 mM) condition, but AME did not affect their insulin secretion levels under low glucose (2.8 mM) condition. Further, we calculated the stimulation index of insulin and found that there were 2.07-fold increase (p < 0.0001) and 1.49-fold increase (p = 0.0014) in 0.5 mg/ml AME group and 1.0 mg/ml AME group respectively when compared to the serum-deprived (BSA only) group (**Figure 3**). The current data supported the protective effect of AME on serum-deprived islets, which peaked at the concentration of 0.5 mg/ml; therefore, the concentration of 0.5 mg/ml was used for subsequent experiments.

### The Protective Effect of AME Is Mediated by Increasing PI3K/Akt and ERK1/2 Expression

**Figure 4:**
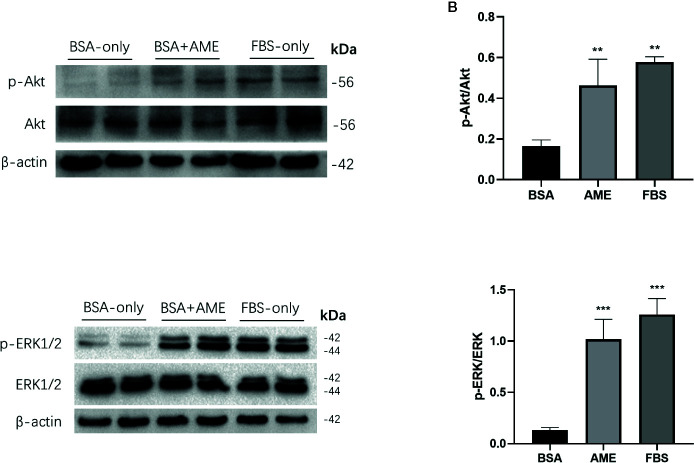
AME increased the phosphorylation of Akt and ERK1/2. **(A, C)** Protein levels of p-Akt, Akt, p-ERK1/2, and ERK1/2 were assessed by Western Blot **(B)**. and **(D)** Quantification of protein levels showed the enhanced expression of Akt and ERK1/2 after AME (0.5 mg/ml) treatment (**p < 0.01 and ***p < 0.001 compared to BSA only group, One-way ANOVA). Results are shown as means ± SD of three independent experiments.

The Ser/Thr kinase Akt (protein kinase B) is essential in PI3K/Akt cascade and in promoting islet β-cell survival (44, 45). Hence, we investigated the effect of AME on Akt in islet cells after 48 h serum-deprivation. Western blot analysis revealed that AME significantly activated Akt phosphorylation compared to serum-deprived islet cells (**Figures 4A, B**). Given that the mitogen-activated protein kinase (MAPK) ERK1/2 can promote islet β-cells survival following extracellular matrix treatment (46), we performed western blot analysis of ERK1/2. Results showed that AME significantly increased the phosphorylation of ERK1/2 compared to the serum-deprivation group (**Figures 4C, D**).

### Significant Increased Expression of Bcl2/Bax Ratio in the Presence of AME

**Figure 5:**
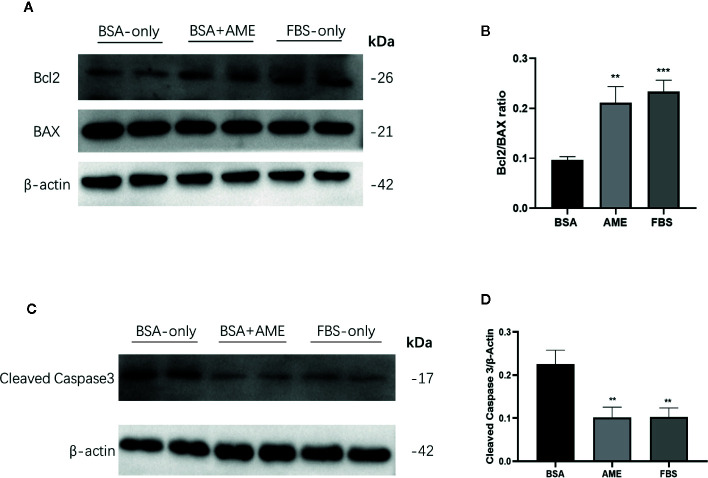
AME increased Bcl2/BAX ratio and decreased the expression of cleaved caspase-3 **(A)**. and **(C)** Protein levels of Bcl2, Bax, cleaved caspase-3, and β-actin were assessed by Western Blot **(B)**. and **(D)** Quantification of protein levels showed the enhanced expression of Bcl2 after AME (0.5 mg/ml) treatment, while there was no significant change of the expression level of Bax (p > 0.05, One-way ANOVA). Further analysis of Bcl2/Bax ratio showed that AME significantly increased Bcl2/Bax ratio of serum-deprived islets (**p < 0.01 and ***p < 0.001 compared to BSA only group, one-way ANOVA). And the expression of cleaved caspase-3 was significantly decreased after AME treatment (**p < 0.01 *vs.* BSA only group, One-way ANOVA). Results are shown as means ± SD of three independent experiments.

To determine the effect of AME on the expression of apoptosis-related proteins, Bcl2, Bax, and cleaved caspase-3 was assessed by western blot analysis. Results showed that AME supplementation significantly increased the expression of Bcl2 compared to serum-deprivation group (p < 0.001), while there was no significant difference in the expression of Bax between AME and serum-deprivation group. Bcl2/Bax ratio was further analyzed and results showed that Bcl2/Bax ratio was significantly increased in AME group compared to serum-deprivation group (p < 0.01) (**Figures 5A, B**). Subsequently, the expression of cleaved caspase-3 was evaluated and results showed that serum-deprivation induced a significantly increased expression of cleaved caspase-3(2.4-fold change) compared to normal FBS group, while supplementation of AME ameliorated this effect (**Figures 5C, D**).

### Non-Fasting Blood Glucose Measurements and Diabetes Reversal Rate Calculation

**Figure 6:**
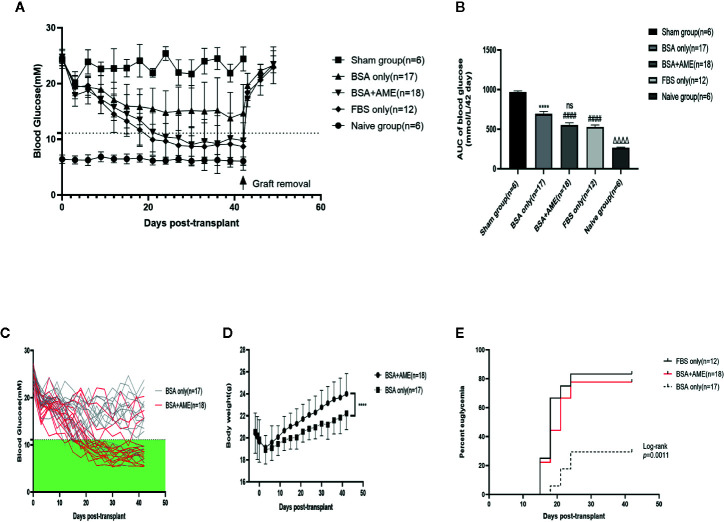
Regulation of recipient blood glucose. Islets were cultured in different conditions (BSA only, BSA + AME [0.5 mg/ml], and FBS only) prior to transplantation. Non-fasting blood glucose was measured every 3 days post-transplantation **(A)**. Non-fasting blood glucose curves of islet recipients and the control group. Data are shown as means ± SD. The dotted line indicates the level of 11.1 mmol/l **(B)**. AUC of non-fasting blood glucose, expressed as mmol/L/42days (^####^p < 0.0001 and ns when BSA + AME group [N = 18] was compared to BSA only [N = 17] and FBS only group [N = 12]; ****p < 0.0001 when BSA only group was compared to the Sham group [N = 6]; ΔΔΔΔp < 0.0001 when Naïve group [N = 6] was compared to BSA + AME and FBS only group, One-way ANOVA) **(C)**. Non-fasting blood glucose of individual recipients in BSA only and BSA + AME group **(D)**. Body weight monitoring of graft recipients in BSA + AME and BSA only group (****p < 0.0001 when BSA + AME group [N = 18] was compared to the BSA only [N = 17] group, Unpaired t-test) **(E)**. The percentage of recipients achieving euglycemia. p = 0.0016 for the *post hoc* comparison of BSA only and BSA + AME group. p = 0.5270 for the *post hoc* comparison of BSA+AME and FBS only group (Log-rank [Mantel-Cox] test).

To further determine the *in vivo* function of cultured islets, we transplanted equal amounts of islets into the renal subcapsular space of STZ-induced diabetic C57BL/6 mice after 48 h cultivation under different conditions (BSA only, BSA + AME, and FBS only). There were no significant differences among the initial non-fasting blood glucose levels of different groups (Sham group, 24.32 ± 1.569 mM; BSA only group, 24.66 ± 1.502 mM; BSA + AME group, 24.72 ± 1.01 mM; FBS only group, 24.46 ± 0.8597 mM) (p > 0.05, **Figure 6A**). After transplantation, the BSA + AME group showed a significant decrease in the area under the curve (AUC) values compared to the BSA only group (554.1 ± 27.59 *vs.* 693.2 ± 30.97 mmol/L/42 day, p < 0.0001, **Figure 6B**). The AUC value of the BSA only group was significantly lower than that of the sham group (693.2 ± 30.97 *vs.* 966.4 ± 16.80 mmol/L/42 day, p < 0.0001, **Figure 6B**), which confirmed the effect of islet transplantation on blood glucose control. However, there was no significant difference between the BSA + AME and FBS only group in AUC values (554.1 ± 27.59 *vs.* 527.7 ± 25.76 mmol/L/42 day, p > 0.05, **Figure 6B**), and the AUC values of the BSA + AME and FBS only group were significantly higher than that of the naive group (554.1 ± 27.59 *vs.* 266.6 ± 6.153 mmol/L/42 day, p < 0.0001; 527.7 ± 25.76 *vs.* 266.6 ± 6.153 mmol/L/42 day, p < 0.0001, **Figure 6B**).

The individual non-fasting blood glucose levels of BSA only and BSA + AME group during the 42-day monitoring were showed in **Figure 6C**. Meanwhile, body weight monitoring showed that graft recipients in BSA + AME group was significantly higher in body weight compared to graft recipients in BSA only group (AUC values, 948.6 ± 11.63 *vs.* 900.2 ± 9.673 g/42 day, p < 0.0001, **Figure 6D**). Additionally, the diabetes reversal rates were significantly different between the BSA only and BSA + AME group (p < 0.05, **Figure 6E**).

### Intraperitoneal Glucose Tolerance Test

**Figure 7:**
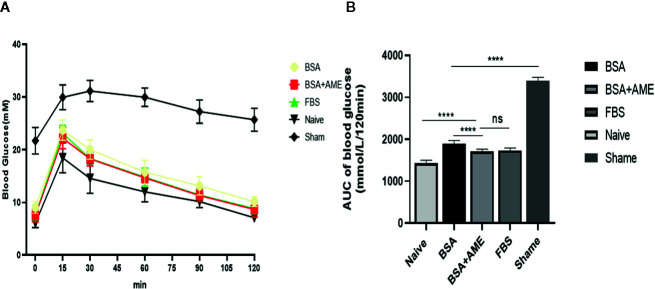
Intraperitoneal glucose tolerance test of islet grafts pre-cultured under different conditions **(A)**. Blood glucose was measured at 0, 15, 30, 60, 90, and 120 min after dextrose I.P. administration. Data points are shown as mean ± SD **(B)**. Areas under the curve of blood glucose tolerance test, expressed as mmol/L/120 min (****p < 0.0001, ns and ****p < 0.0001 when BSA + AME group [N = 14] was compared to BSA only [N = 5], FBS only [N = 10], and Naïve group [N = 6], respectively. ****p < 0.0001 when BSA only group was compared to the Sham group [N = 6] One-way ANOVA).

As shown in **Figure 7**, the AUC value of the intraperitoneal glucose tolerance test of the BSA + AME group was significantly lower than that of the BSA only group (1,712 ± 51.39 *vs.* 1,896 ± 72.99 mmol/L/120 min, p < 0.0001), which indicated that BSA + AME cultured islet graft presented a greater function of blood glucose control than BSA only cultured islet graft. However, there was no significant difference between the AUC values of the BSA + AME and FBS only group (1,712 ± 51.39 *vs.* 1,735 ± 55.15 mmol/L/120 min, p > 0.05).

### Histological Staining and Graft Insulin Content of the Islet Grafts

**Figure 8:**
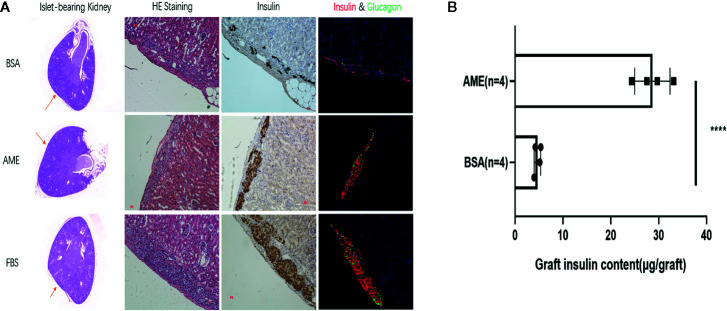
Histological analysis of islet graft and islet graft insulin content assay **(A)**. HE, immunohistochemistry, and immunofluorescence staining of islet grafts. Scale bar: 20 μm **(B)**. Islet grafts were harvested and homogenized to obtain the total insulin content of the grafts (****p < 0.0001 when BSA + AME [N = 4] group was compared to BSA only [N = 4], Unpaired t-test).

Islet grafts were harvested and processed to histological analysis. Representative images of the HE staining, immunohistochemistry staining for insulin, and immunofluorescence staining for insulin and glucagon of islet grafts were displayed in **Figure 8A**, which indicated the survival of islet grafts under different pre-cultured conditions.

To quantitatively analyze the islet graft survival, graft insulin content assay was conducted. As shown in **Figure 8B**, the graft insulin content of the BSA + AME group (n = 4) was significantly higher compared to the BSA only group (n = 4) (28.69 ± 3.681 *vs.* 4.681 ± 0.6876 μg/graft, p < 0.0001), which suggested that AME supplementation during serum-deprived culture maintained islet viability and promoted islet graft survival. However, there was no significant difference between BSA + AME and FBS only group (28.69 ± 3.681 *vs.* 29.09 ± 1.008 μg/graft, p > 0.05).

### Concentrations of Growth Factors in Amniotic Membrane Extract

In an attempt to elucidate the possible composition of AME, several growth factors reported to be beneficial for islet survival were assayed by commercially available ELISA kits. To assess the stability of AME, freshly prepared AME was assayed for the concentrations of growth factors pre- and post-storage at −80°C. Results showed that the concentrations of the growth factors tested were stable in the first month of storage at −80°C. Meanwhile, the concentrations of TIMP-1, EGF, HGF, and IL-1RA were further tested by ELISA kits in freshly prepared AME and in cryopreserved AME that was prepared 3 and 6 months ago respectively. The concentration of AME was 1.0 mg/ml in all samples tested by ELISA. Results showed that the concentrations of growth factors tested declined in cryopreserved AME, however the differences were not significant (*p* > 0.05) (**Table 1**).

**Table 1:** Concentrations of growth factors in fresh and cryopreserved AME.

Fresh AME			Cryopreserved AME		
Growth factors	Pre-Storage	Post-storage at -80°C			
		7 days	1 month	3 months	6 months
TIMP-1 (ng/ml)	51.57±23.05	48.80±19.38	44.29±13.19	45.27±13.85	42.94±16.24
EGF (pg/ml)	199.93±14.03	202.30±25.22	197.20±22.42	198.97±10.71	179.73±10.50
HGF (pg/ml)	3035.02±212.33	2957.10±116.56	3004.13±279.92	2903.95±111.35	2764.74±151.51
IL-1RA (pg/ml)	2094.19±273.72	2172.98±201.22	2023.68±270.06	1964.08±156.59	1889.64±221.50

All data are presented as mean±SD.

## Discussion

Pretransplant islet culture has been adopted as a routine step to induce immunosuppressive therapy in the recipient, enable islet quality control and islet shipment and cross-center transportation, increase islet purification, and minimize post-transplantation nonspecific inflammation (8–10, 47). However, isolated islets were stressed, and islet function was impaired during the *in vitro* cultivation period, which may result in unsatisfactory transplant outcomes. Various efforts have been made to preserve islet mass and function before transplantation, including supplementation of additives like green tea extract (48) and liraglutide (49); use of scaffolds or extracellular matrix (50–52), and modulation of the culture temperature and gas environment (53, 54). To the best of our knowledge, this is the first study that demonstrated how using human amniotic membrane extract as medium supplementation to improve islet viability and function. As described in the results, AME supplementation significantly improved islet viability and GSIS function, as well as reduced islet apoptosis rate and percent of islet loss after 48 h serum-deprived culture. And the PI3K/Akt and MAPK/ERK pathways were involved in the protective effect of AME on islet as indicated by the increased expression of phosphorylated Akt and ERK1/2. Further, we found that AME supplementation increased the Bcl2/BAX ratio in serum-deprived islet, and with decreased expression of cleaved caspase-3. Finally, we found that supplementation of AME in islet culture medium improved the engraftment efficiency of the cultured islet. Within the 6-week observation period, recipients in the AME-treated-islet group showed a significant improvement in blood glucose control and graft insulin release function compared to the basal control group. In addition, there was a significant difference between the BSA+AME group and BSA only group in graft survival, which illustrated that AME-treated islets possessed a greater potential to survive and regulate recipient blood glucose compared to serum-deprived islets post-transplantation.

The amniotic membrane (AM) is a natural biomaterial that has been used in the treatment of various diseases, such as ophthalmology and burns disorder (25, 55). Previous studies have focused on the cells and scaffold from AM. And it has been reported that human amniotic epithelial cells (AECs) possess localized immune privilege *in vitro*, thus having a potential implication for islet transplantation (56). Another study engineered insulin-producing organoids from islet and amniotic epithelial cells, which markedly enhanced engraftment viability and graft function (57). Further, it was reported that by co-infusing human amniotic epithelial cells and islets into decellularized AM and then transplanting it into diabetic mice, it is possible to achieve good engraftment outcomes (58). These studies implicate the application potential of AME in islet transplantation. Recently, increasing studies have explored the properties of AME due to its extensive biomolecules and low immunogenicity, and result shown that AME promoted limbal stem cell proliferation and corneal epithelium healing (20), protected primary human corneal epithelial (HCE) cells and human limbal cells from oxidative stress (22). More recently, it was reported that amniotic membrane extract protected H9c2 cardiomyoblasts against hypoxia-induced apoptosis (23, 59). Taken together, these suggest that AME has cytoprotective effects and might serve as cytoprotective agents in islet transplantation.

The PI3K/Akt pathway plays a key role in mediating cell proliferation, survival, and metabolism (60). It was reported that growth factors like hepatocyte growth factor and epidermal growth factor could activate PI3K/Akt signaling pathway, leading to cell growth and survival (26, 61). In particular, studies have shown that activation of Akt has a pivotal role in isolated islet survival during *in vitro* culture (44, 45, 62–64). Another signal pathway involved in the regulation of isolated islet survival is the MAPK pathway (65). The MAPK family includes three major subgroups: extracellular signal-regulated kinases (ERKs), c-Jun N-terminal kinases (JNKs), and p38 MAPK (p38). It was reported that the extracellular matrix derived from 804G cells protected islet β-cells against apoptosis *via* the activation of Akt and ERK (46). Our previous study also showed that increased ERK activation had a protective effect on islet β-cells (66). Considering that AME is a mixture of growth factors and extracellular matrix components, we inferred that the protective effect of AME on islet might involve PI3K/Akt and MAPK/ERK pathway, which is also consistent with the results of our study.

Nevertheless, there are some limitations to the present study. Firstly, we characterized AME by measuring the concentrations of several growth factors reporting to have prosurvival effects on islet. However, it was reported that AME at high concentrations had a negative impact on cell growth (20), thus suggesting a potential presence of some components that could impair cell viability at certain levels. Our data showed that AME no longer protected islet at the concentration of 1.5 mg/ml, implicating that there existed some components harmful to islet at high concentrations. Therefore, it is necessary to define the exact constituents of AME in our further investigation. Then, primary islets isolated from C57BL/6 mice were used in this study, which may differ from primary human islets. With some ethical and technical issues, we could not get access to human islets currently. Finally, considering AME was a mixture, it was not suitable to be co-transplanted with islets into renal subcapsular space nor portal vein, which hindered the observation of the AME effects on the islet *in vivo*. While, we are investigating the possibility of AME combined with tissue engineering during islet transplantation in our ongoing experiments.

In conclusion, the present study demonstrated that supplementation of AME during serum-deprived pretransplant culture improved isolated islet survival and function, which consequently improved islet engraftment outcomes. The PI3K/Akt and MAPK/ERK had an essential role in the protective effect of AME. Therefore, our results suggest that AME might serve as a potentially effective medium supplement in the pretransplant culture of islets isolated for clinical transplantation.

## Data Availability Statement

The raw data supporting the conclusions of this article will be made available by the authors, without undue reservation, to any qualified researcher.

## Ethics Statement

The studies involving human participants were reviewed and approved by Ethical Committee of the First Affiliated Hospital of China Medical University. The patients/participants provided their written informed consent to participate in this study. The animal study was reviewed and approved by Animal Care and Use Committee of China Medical University.

## Author Contributions

Conception and design of the study: ZY and JZ. Acquisition of data: ZY, TG, and FL. Analysis and interpretation of data: ZY and JL. Drafting or revising the manuscript: ZY, XL, CZ, NS, JL, and JZ. All authors contributed to the article and approved the submitted version.

## Funding

This work was supported by the program of Liaoning provincial science and technology department (Grant no.2017225031).

## Conflict of Interest

The authors declare that the research was conducted in the absence of any commercial or financial relationships that could be construed as a potential conflict of interest.
